# Diagnostic Yield of Phenotype-Guided Genetic Testing in Infertility: A Five-Year Retrospective Study from a Tertiary Referral Cohort

**DOI:** 10.3390/diagnostics16060896

**Published:** 2026-03-18

**Authors:** Kristina Aleknavičienė, Marius Šukys, Živilė Žemeckienė, Birutė Žilaitienė, Rasa Ugenskienė

**Affiliations:** 1 Department of Genetics and Molecular Medicine, Medical Academy, Lithuanian University of Health Sciences, 44307 Kaunas, Lithuania; 2 Institute of Endocrinology, Medical Academy, Lithuanian University of Health Sciences, 44307 Kaunas, Lithuania

**Keywords:** diagnostic yield, infertility, genetic testing, karyotype, Y chromosome microdeletion

## Abstract

**Background/Objectives**: To evaluate the diagnostic yield of phenotype-guided cytogenetic and targeted molecular genetic testing in patients referred for infertility and reproductive disorders within a tertiary medical genetics referral pathway. **Methods**: This retrospective study included 900 consecutive patients (473 males, 427 females) referred to a tertiary medical genetics center between January 2020 and December 2024. Conventional karyotyping was performed in all patients. Additional targeted molecular tests were applied based on clinical indication: Y chromosome microdeletion analysis in azoospermia or oligospermia, *CFTR* sequencing in suspected congenital bilateral absence of the vas deferens, and *F2*/*F5* genotyping in recurrent pregnancy loss (RPL). Diagnostic yield was analyzed in a predefined subgroup of 566 patients with RPL, unexplained infertility, azoospermia, or oligospermia; remaining referrals were included in descriptive cytogenetic analyses only. **Results**: Chromosomal abnormalities were identified in 3.22% (29/900) of the total cohort and in 5.12% (29/566) of the diagnostic-yield cohort. Targeted testing yields were 3.75% (6/160) for Y chromosome microdeletions, 9.38% (3/32) for *CFTR* variants, and 3.31% (4/121) for *F2*/*F5* variants. Diagnostic yield varied markedly by phenotype, being highest in azoospermia (33.3%), followed by oligospermia (6.6%), RPL (5.3%), and unexplained infertility (3.1%). In unexplained infertility, all chromosomal abnormalities were detected in female patients. **Conclusions**: In a tertiary referral setting, phenotype-guided genetic testing provides the greatest diagnostic value in well-defined infertility phenotypes, particularly azoospermia. Lower yields in other referral groups support a targeted, indication-based approach to genetic evaluation and highlight the need for advanced genomic strategies in persistently unexplained cases.

## 1. Introduction

Infertility is a multifactorial condition affecting approximately 17.5% of couples of reproductive age worldwide [1]. It presents a significant clinical and psychological burden and often requires a comprehensive evaluation to determine its underlying causes. While numerous environmental, hormonal, anatomical, and infectious factors contribute to infertility, a substantial proportion of cases—particularly those involving severe male infertility or recurrent pregnancy loss—are suspected to have a genetic basis [2,3,4].

Genetic abnormalities may impair fertility via diverse mechanisms, including chromosomal aneuploidies, structural rearrangements (e.g., balanced/unbalanced translocations), single-gene variants, microdeletions, copy-number variants, and imprinting defects. Karyotyping has long been a cornerstone of genetic diagnostics in infertility, especially in patients with azoospermia, severe oligospermia, or recurrent miscarriage, where it can reveal sex-chromosome aneuploidies (e.g., 47,XXY), large rearrangements, or mosaicism. Complementary targeted molecular tests are commonly employed: Y chromosome microdeletion assays covering AZF regions (AZFa/b/c), *CFTR* sequencing for congenital bilateral absence of the vas deferens (CBAVD), and testing for thrombophilia-associated variants (e.g., *F2*, *F5*) in recurrent pregnancy loss [2,5].

Among these, Y chromosome microdeletions are one of the best-recognized genetic contributors to male infertility. AZFc deletions are frequently associated with non-obstructive azoospermia or severe oligospermia, whereas AZFa/AZFb deletions often result in more profound spermatogenic failure and substantially poorer prospects for sperm retrieval. While conventional cytogenetic and AZF testing identify clinically relevant defects in a meaningful minority of patients, many patients remain without a molecular diagnosis, highlighting the limits of these approaches and the value of broader genomic analyses [5].

Despite guidance from professional societies, standardized genetic testing pathways are unevenly implemented across regions. Practical barriers include variable access to specialized laboratories, differences in reimbursement, and limited evidence that specific test results consistently alter clinical management or outcomes. Accordingly, many clinics still employ a stepwise algorithm—karyotype first; Y-microdeletion and *CFTR* testing as indicated; then expanded molecular sequencing in select cases—rather than upfront comprehensive genomic profiling [3,5]

In Lithuania, the use of genetic testing within infertility evaluations has increased in recent years; however, comprehensive data on diagnostic yield remain limited, and no unified national protocol has been established. This context underscores the need for a systematic assessment of current practices.

From a diagnostic perspective, understanding the yield and limitations of established cytogenetic and targeted molecular tests across infertility phenotypes is essential for optimizing test selection and resource utilization in clinical genetics laboratories. Accordingly, this study aims to evaluate the diagnostic yield of phenotype-guided cytogenetic and targeted molecular testing—including karyotyping, Y chromosome microdeletion analysis, *CFTR* sequencing, and *F2*/*F5* variant testing—in patients referred for infertility and reproductive disorders over a five-year period at a tertiary medical genetics center in Lithuania. The findings are intended to clarify the current clinical utility of these tests and to inform decisions regarding the appropriate positioning of advanced genomic technologies (e.g., exome or genome sequencing) within specialized infertility care pathways.

Data on the diagnostic performance of infertility-related genetic testing remain scarce in Lithuania, and standardized national recommendations for genetic evaluation in infertility have not yet been formally established.

## 2. Materials and Methods

### 2.1. Study Group

This retrospective study included patients referred to the Department of Genetics and Molecular Medicine at the Hospital of the Lithuanian University of Health Sciences, Kauno klinikos, between January 2020 and December 2024 for genetic consultation related to infertility or reproductive disorders. Eligible participants were identified through institutional clinical records and included all individuals referred for genetic evaluation associated with infertility, recurrent pregnancy loss, or other reproductive conditions during the study period.

A total of 900 patients met the inclusion criteria and were included in the overall cohort. Patients were referred from reproductive medicine specialists, endocrinologists, and other clinicians involved in infertility care. Referral indications included azoospermia, oligospermia, unexplained infertility, recurrent pregnancy loss, secondary infertility, hypogonadotropic hypogonadism, primary or secondary amenorrhea, ovarian insufficiency, and other reproductive disorders.

This study was approved by the Kaunas Regional Biomedical Research Ethics Committee (permission No. BE-2-130; approval date: 18 December 2024). The study used secondary, fully anonymized clinical and genetic data; therefore, informed consent was not required.

In this study, patients were categorized based on their initial reasons for admission, allowing us to assess them within distinct subgroups of reproductive disorders. Patients were divided into three main subgroups based on their primary reproductive diagnoses at the time of referral:

1. Azoospermia/Oligospermia Group—This group included male patients diagnosed with either azoospermia (complete absence of sperm in the ejaculate) or oligospermia (reduced sperm count), based on semen analysis according to WHO criteria.
2. Unexplained Infertility Group—This subgroup consisted of patients (both male and female) in whom no definitive cause of infertility was identified after standard clinical, hormonal, and imaging evaluations.
3. Recurrent Miscarriages Group—This group included couples with a history of two or more consecutive pregnancy losses before 20 weeks of gestation, without an identified anatomical, infectious, or hormonal cause.

All patients underwent genetic consultation and standardized genetic evaluation as part of an indication-based diagnostic evaluation. Genetic testing practices remained consistent throughout the study period, with specific tests selected according to clinical guidelines, physician judgment, and the patient’s presenting reproductive disorder. Tests were tailored to the suspected underlying etiology within each subgroup, ensuring that each individual received an appropriate, indication-based diagnostic assessment. Genetic testing indications were based on international clinical practice recommendations, including guidelines from the European Academy of Andrology (EAA) and the European Society of Human Reproduction and Embryology (ESHRE), as well as standard clinical genetics practice at our institution.

Karyotyping was performed for all patients, regardless of sex or clinical subgroup, using G-banding analysis on peripheral blood lymphocytes. Chromosomal abnormalities were described according to the International System for Human Cytogenomic Nomenclature (ISCN).

Y chromosome microdeletion analysis was conducted in male patients from the azoospermia/oligospermia group using multiplex PCR to detect deletions in the AZFa, AZFb, and AZFc regions, targeting genetic causes of spermatogenic failure.

*CFTR* gene sequencing was carried out in cases of azoospermia in which congenital bilateral absence of the vas deferens (CBAVD) was suspected based on clinical and imaging findings. Full sequencing of the *CFTR* coding regions and evaluation of common pathogenic variants were performed.

*F2* (prothrombin c.*97G>A) and *F5* (factor V Leiden c.1601G>A) variant analysis was performed using real-time PCR in female patients from the recurrent miscarriage group to assess potential hereditary thrombophilia as a contributing factor to pregnancy loss.

For diagnostic yield analysis, patients were grouped by their primary clinical indication at referral. Diagnostic yield calculations were restricted to phenotypes with established indications for the evaluated tests, including azoospermia, oligospermia, unexplained infertility, and recurrent pregnancy loss. Patients referred for secondary infertility, hypogonadotropic hypogonadism, primary or secondary amenorrhea, ovarian insufficiency, and other heterogeneous reproductive disorders were included in the overall cohort description but excluded from subgroup diagnostic yield calculations because the targeted molecular tests evaluated in this study were not routinely implemented for these phenotypes in our diagnostic pathway. In these patients, cytogenetic testing was primarily performed to exclude major chromosomal abnormalities. Genetic evaluation of hypogonadotropic hypogonadism in our center typically involves broader genomic approaches such as exome sequencing, which were outside the scope of the present analysis. Genetic tests were carried out at the Department of Genetics and Molecular Medicine, following standardized laboratory protocols and quality control procedures.

### 2.2. Methods

#### 2.2.1. Karyotyping

Peripheral blood lymphocyte cultures were prepared and analyzed for chromosomal abnormalities using standard cytogenetic techniques. Approximately 0.5 mL of heparinized peripheral blood was cultured in 9.5 mL of complete growth medium (Capricorn Scientific, Ebsdorfergrund, Germany) supplemented with phytohemagglutinin (PHA) to stimulate lymphocyte division. Cultures were incubated for 72 h at 37 °C in two parallel setups: one for standard lymphocyte culture and one for high-resolution banding using the methotrexate-thymidine cell culture synchronization method. Colcemid (Capricorn Scientific, Germany) was added 30 min before fixation to arrest cells in metaphase.

Following incubation, cells were treated with 0.075 M KCl hypotonic solution (Capricorn Scientific, Germany) and subsequently fixed with fixative solution (methanol/acetic acid (3:1)). Multiple fixation steps were performed until the cell pellet appeared clear. Fixed cell suspensions were dropped onto pre-cleaned glass slides to obtain well-spread metaphase preparations, which were then aged overnight at 56 °C and stained using the GTG-banding (G-banding with trypsin and Giemsa) technique. Slides were initially treated with trypsin solution for 45 s, washed in FBS and PBS (Gibco, Waltham, MA, USA) solutions, stained with GURR buffer-Giemsa solution (Gibco, US) for 16 min, and finally rinsed with water.

A minimum of 15 metaphases were analyzed per individual with a normal karyotype, whereas 20 cells were examined in cases where structural or numerical abnormalities were detected. In cases of suspected mosaicism, at least 50 metaphases were evaluated. Mosaicism was suspected in cases where preliminary metaphase evaluation suggested cell line variability or when clinical findings raised suspicion of chromosomal mosaicism. Microscopic analysis was performed using a computer-assisted karyotyping system with digital imaging software GenASIs Version 8. Karyotypes were described and reported according to the International System for Human Cytogenomic Nomenclature (ISCN, 2020 [6]).

#### 2.2.2. Y Microdeletions Analysis

Genomic DNA was extracted from peripheral blood leukocytes using standard procedures (QIAsymphony DNA Midi Kit (Qiagen, Hilden, Germany)). Y chromosome microdeletion analysis was performed in male patients with azoospermia or oligospermia, following the European Academy of Andrology (EAA) and the European Molecular Genetics Quality Network (EMQN) best-practice guidelines.

Multiplex PCR assays were carried out using the AZF v2 and AZF Extension commercial kits (Devyser, Stockholm, Sweden), which amplify sequence-tagged sites (STSs) specific for the AZFa (sY84, sY86), AZFb (sY127, sY134), and AZFc (sY254, sY255) regions, as well as additional markers (sY82, sY83, sY1065, sY88, sY105, sY121, sY1192, sY153, sY160) to confirm partial or complex deletions. Internal controls included sY14 (SRY) and ZFX/ZFY to verify amplification and DNA integrity.

Amplified fragments were separated by capillary electrophoresis on an Applied Biosystems 3500 Genetic Analyzer (Applied Biosystems, Foster City, CA, USA). Data were analyzed with GeneMapper software 6 (Thermo Fisher Scientific, Waltham, MA, USA).

#### 2.2.3. *CFTR* Variant Analysis Using Sanger Sequencing

Genomic DNA from peripheral blood (QIAsymphony DNA Midi Kit (Qiagen, Hilden, Germany)) was amplified by PCR using AmpliTaq Gold 360 (Thermo Fisher Scientific, Waltham, MA, USA) master mix with gene-specific primer pairs carrying M13 universal tails. Thermal cycling followed a touchdown protocol, yielding amplicons for bidirectional sequencing.

Post-PCR cleanup was carried out with Exonuclease I and alkaline phosphatase (Thermo Fisher Scientific, Waltham, MA, USA). Cycle sequencing used BigDye Terminator v3.1 (Thermo Fisher Scientific, Waltham, MA, USA) chemistry with M13F/M13R primers and 5× sequencing buffer. Sequencing products were purified with BigDye XTerminator Purification Kit (Thermo Fisher Scientific, Waltham, MA, USA) and resolved by capillary electrophoresis on an Applied Biosystems 3500 Genetic Analyzer (Applied Biosystems, Foster City, CA, USA).

Raw chromatograms were processed with the manufacturer’s 3500 Series Data Collection/Sequencing software 3 and aligned to the RefSeq transcript NM_000492.4 (*CFTR*). Variants were described according to HGVS nomenclature at the transcript level. Positive findings were confirmed by bidirectional reads within the same run.

#### 2.2.4. *F2*/*F5* Common Variants Analysis Using Real-Time PCR

Detection of the *F2* and *F5* variants was performed by allele-specific real-time PCR (TaqMan SNP genotyping). Genomic DNA extracted from peripheral blood was analyzed using the Type-it Fast SNP Probe PCR Master Mix (Qiagen, Hilden, Germany) with commercially available TaqMan primer-probe assays (Thermo Fisher Scientific, Waltham, MA, USA) specific for each variant.

Fluorescence was measured in the FAM (wild-type allele) and VIC (variant allele) channels, and genotype calling was performed by scatter plot (allelic discrimination) analysis using the Rotor-Gene Q software Pure Detection 2.3.5. Each sample was automatically assigned as homozygous wild-type, heterozygous, or homozygous mutant according to amplification signal clustering. All results were reviewed and exported as Scatter Analysis Reports and verified manually.

#### 2.2.5. Data Analysis and Statistics

Descriptive statistics were used to summarize patient demographics, referral categories, and the distribution of genetic findings. All data processing and tabulation were performed using MS Office Excel.

Diagnostic yield was defined as the proportion of patients in whom genetic testing identified a pathogenic or likely pathogenic finding considered clinically relevant for the investigated infertility phenotype. For cytogenetic analysis, this included chromosomal aneuploidies and structural rearrangements with known reproductive significance. For molecular testing, this included Y chromosome microdeletions and pathogenic *CFTR* variants associated with CBAVD. Heterozygous *F2* and *F5* variants were reported separately because their contribution to recurrent pregnancy loss remains debated, and they were not considered definitive causal findings.

## 3. Results

### 3.1. Demographic Data

**Figure 1:**
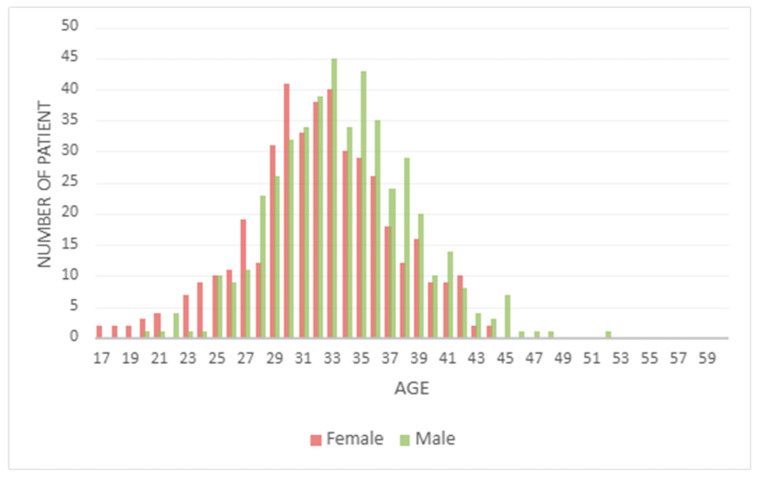
Age Distribution of the patients. The median age at the time of referral was found to be 33 years for male patients and 32 years for female patients.

In the total cohort, the age distribution of the patients is shown in Figure 1. The median age at the time of referral was found to be 33 years for male patients and 32 years for female patients.

The most common reasons for referral to genetic testing were recurrent miscarriages (25.3%), secondary infertility (26.3%), and oligospermia (13.6%). Recurrent miscarriages accounted for 28.3% of referrals among female patients and 22.6% among males, the latter representing partners of affected women undergoing karyotype evaluation as part of couple-based infertility assessment. Secondary infertility was identified in 32.1% of females and 21.1% of males. Unexplained infertility was also a significant cause for referral, observed in 17.8% of the total cohort.

Among male patients, the leading referral indications were oligospermia (22.4%), recurrent miscarriages (22.6%), and azoospermia (11.4%). In contrast, female patients were most frequently referred for recurrent miscarriages (28.3%), unexplained infertility (19.9%), and primary or secondary amenorrhea (7.5%).

Less common indications across the cohort included hypogonadotropic hypogonadism (0.9%), ovarian insufficiency (0.8%), and a small group categorized as Other Reproductive Disorders (5.6%), which included patients with undefined or non-specific reproductive issues.

A detailed breakdown of referral reasons and their distribution by gender is provided in Table 1.

Karyotyping was performed for all 900 patients in the cohort, including 473 males (52.6%) and 427 females (47.4%). Patients referred for secondary infertility, hypogonadotropic hypogonadism, primary or secondary amenorrhea, ovarian insufficiency, and other reproductive disorders (*n* = 334) were included only in the general cytogenetic evaluation. None of the individuals in these subgroups showed chromosomal abnormalities. They were not included in the final study group used for diagnostic yield analysis.

The study group, from which diagnostic yield was calculated, consisted exclusively of patients referred for recurrent miscarriages, unexplained infertility, azoospermia, and oligospermia (subgroups highlighted in gray in Table 1), totaling 566 individuals.

**Figure 2:**
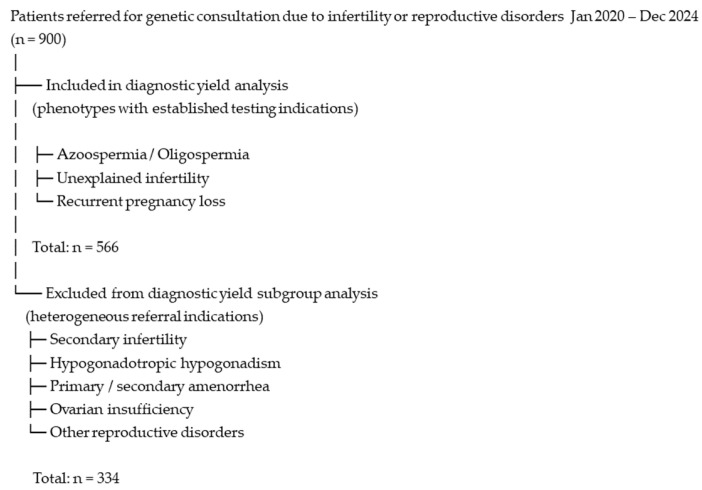
Flow diagram of patient inclusion and subgroup selection for diagnostic yield analysis.

The selection of patients included in the diagnostic yield analysis is illustrated in Figure 2.

### 3.2. Diagnostic Yield of Cytogenetic and Targeted Molecular Testing

**Figure 3:**
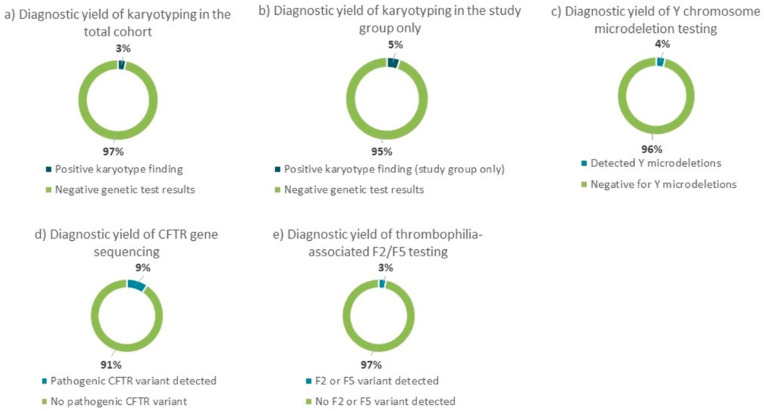
Diagnostic yield of genetic testing across different patient groups. (**a**) Diagnostic yield of karyotyping in the total cohort. (**b**) Diagnostic yield of karyotyping in the study group only (patients with recurrent miscarriage, unexplained infertility, azoospermia, oligospermia, and aspermia). (**c**) Diagnostic yield of Y chromosome microdeletion testing in males with azoospermia or oligospermia. (**d**) Diagnostic yield of *CFTR* gene sequencing in patients with suspected CBAVD. (**e**) Diagnostic yield of *F2*/*F5* mutation testing in patients with recurrent miscarriage. Percentages shown in the figure are rounded to the nearest whole number for visualization purposes.

A total of four genetic tests were performed as part of the standard diagnostic evaluation in referred patients. Karyotyping, which was performed on all patients, revealed chromosomal abnormalities in 3.22% (29/900) of the total cohort. However, when considering only the subset of patients with specific reproductive disorders—including recurrent miscarriages, unexplained infertility, azoospermia, and oligospermia—the prevalence of abnormal karyotypes increased to 5.12% (26/566), with 94.88% of patients showing normal results. Y chromosome microdeletion analysis, conducted in males with azoospermia or oligospermia, detected deletions in 3.75% (6/160) of cases. *CFTR* gene sequencing, performed in patients with suspected CBAVD, identified pathogenic variants in 9.38% (3/32) of those tested. Lastly, *F2* (prothrombin c.*97G>A) and *F5* (factor V Leiden c.1601G>A) variant testing, conducted in patients with recurrent miscarriage, revealed inherited thrombophilia-related variants in 3.31% (4/121) of cases. The remaining patients in each testing group showed no detectable genetic abnormalities. These findings are summarized in Figure 3.

Diagnostic yield varied across different clinical subgroups. Among patients with azoospermia, a genetic diagnosis was achieved in 33.3% of cases, including abnormalities detected by karyotyping, Y chromosome microdeletion analysis, and *CFTR* gene sequencing. In the oligospermia group, 6.6% of patients had a positive genetic result, primarily through karyotyping and Y chromosome testing. For the recurrent miscarriage group, 5.3% of patients were found to have genetic abnormalities, with results coming from both karyotyping and *F2*/*F5* thrombophilia-related variant analysis. In the unexplained infertility group, a diagnostic yield of 3.1% was observed, based entirely on karyotyping results. Additional analysis of the unexplained infertility subgroup revealed that all detected chromosomal abnormalities were found in female patients, suggesting a predominantly maternal contribution to the genetic etiology in these couples. The identified karyotype changes included structural and numerical X-chromosome anomalies. A detailed overview of diagnostic findings by clinical subgroup is presented in Table 2.

A total of 42 distinct genetic abnormalities were identified across the study cohort, including both chromosomal (*n* = 29) and molecular variants (*n* = 13). Chromosomal anomalies were observed in patients with Klinefelter syndrome (47,XXY; *n* = 9), mosaic Klinefelter (*n* = 1), and various forms of Turner syndrome or Turner variants, including classical monosomy X, mosaic Turner, deletions on the X chromosome, and isochromosome X. In addition, structural chromosomal abnormalities were identified, including Robertsonian translocations (*n* = 3), reciprocal translocations (*n* = 5), and other rare structural rearrangements involving the Y chromosome and marker chromosomes (*n* = 5). During data analysis, polymorphic variants were considered normal karyotypes and excluded.

**Figure 4:**
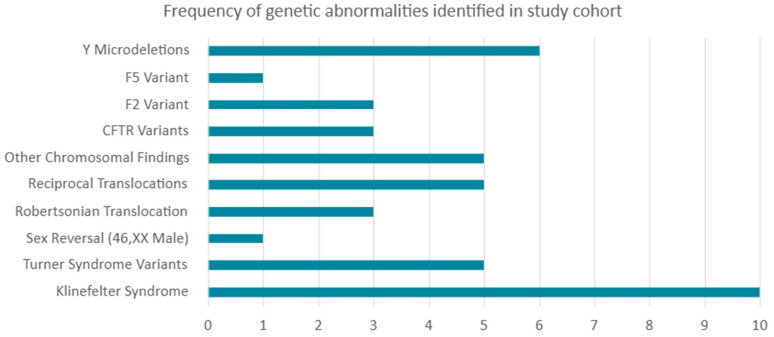
Frequency of genetic abnormalities identified in the study cohort.

Molecular testing revealed *CFTR* gene variants in three patients: two carried the common intron 9 polymorphism c.1210-33_1210-6GT[12]T[5] in combination with the c.1521_1523del (p.Phe508del) deletion, and one carried the same intron variant together with the c.2052dupA (p.Gln685fs) variant. *F2* and *F5* thrombophilia-associated variants were identified in four patients: three with the prothrombin c.*97G>A (*F2*) variant and one with factor V Leiden c.1601G>A (*F5*) variant. All identified carriers were heterozygous, and no double heterozygosity was observed. Y chromosome microdeletions affecting the AZFc (b2/b4) region were detected in six patients with azoospermia or oligospermia. All identified variants are listed in Table 3, and the frequency of these genetic abnormalities within the study cohort is illustrated in Figure 4.

## 4. Discussion

This study provides one of the first systematic assessments of infertility-related genetic testing outcomes within the Lithuanian healthcare system.

This five-year retrospective study evaluated the diagnostic yield of phenotype-guided genetic testing within a tertiary medical genetics referral pathway for infertility and reproductive disorders. Among 900 consecutively referred individuals, diagnostic-yield analyses were focused on 566 patients with recurrent pregnancy loss (RPL), unexplained infertility, azoospermia, or oligospermia, while other reproductive phenotypes were included only in descriptive cytogenetic analyses. Importantly, this study was not designed to estimate population-level prevalence of genetic abnormalities, but rather to assess the performance of commonly used cytogenetic and targeted molecular tests in a real-world, referral-based clinical setting. A limitation is the referral-based nature of the cohort, as patients were evaluated in a tertiary medical genetics center. Consequently, the study population likely represents a selected group with higher clinical suspicion of genetic causes compared with general infertility populations.

Our overall diagnostic yield of approximately 3.22% (chromosomal abnormalities on karyotyping within the study group) aligns with the literature, which reports similar rates of chromosomal causes in infertility cohorts. Earlier studies have reported karyotype abnormality rates in patients with infertility and reproductive disorders ranging between 2.37% and 18.5% [7,8,9,10,11]. Despite technological progress in genetics, traditional cytogenetic testing, such as karyotyping, remains a crucial component of infertility diagnostics. The detection of chromosomal abnormalities—including structural rearrangements such as Robertsonian or reciprocal translocations, and mosaic chromosomal anomalies—has clear clinical significance and can directly impact reproductive outcomes and management decisions. While next-generation sequencing (NGS) enhances molecular resolution, karyotyping continues to provide indispensable information on large-scale chromosomal changes that would otherwise remain undiagnosed if only short-read sequencing-based methods were applied.

Notably, we observed a 33.3% diagnostic yield among azoospermia cases, consistent with prior findings that karyotype anomalies, Y chromosome microdeletions, and *CFTR* mutations contribute up to 25–35% of such cases [12,13,14]. Similarly, our detection rate of *CFTR* variants in patients with suspected CBAVD was 9.38%, which closely aligns with the 9.9% prevalence reported by Stuppia and colleagues in males with non-obstructive azoospermia [15]. The relatively high diagnostic yield observed in azoospermic males emphasizes the importance of combining karyotyping, Y chromosome microdeletion analysis, and *CFTR* testing in this subgroup. However, the majority of azoospermic men in our cohort remained without a molecular diagnosis, highlighting both the biological heterogeneity of male infertility and the limitations of currently applied targeted testing approaches. These findings highlight the potential value of broader genomic approaches, such as exome sequencing, in research settings aimed at improving diagnostic resolution in unresolved infertility cases. Recent exome-based studies in men with idiopathic spermatogenic failure have reported additional molecular diagnoses in approximately 10–12% of cases, including pathogenic or likely pathogenic variants in genes such as *NR5A1*, *TEX11*, and *M1AP*. Meanwhile, exome-based analyses have also highlighted the relevance of *DDX3Y*, a gene within the AZFa region, for non-obstructive azoospermia [16,17,18]. These observations suggest that broader sequencing may improve diagnostic resolution in selected unresolved phenotypes, although such methods were not evaluated in the present cohort.

In contrast, substantially lower diagnostic yields were observed in patients with oligospermia, unexplained infertility, and RPL. These findings underscore the limited utility of indiscriminate genetic testing in selected referral populations and emphasize the importance of phenotype-driven test selection. In women with recurrent miscarriage, our detection of *F2* or *F5* thrombophilia-associated variants in 3.31% of cases corresponds with the lower end of reported prevalence ranges in similar cohorts [19]. While studies have shown associations between Factor V Leiden (*F5* c.1601G>A) and prothrombin (*F2* c.*97G>A) variants and RPL, the clinical utility of non-selective thrombophilia testing remains limited. According to large-scale population datasets such as gnomAD v4, the *F2* c.*97G>A variant has an allele frequency of 0.01138, including 136 homozygous individuals, while the *F5* c.1601G>A variant occurs at a frequency of 0.02143, with 450 homozygotes reported [20]. These relatively high carrier frequencies in the general population suggest that, although these variants confer an increased risk of venous thromboembolism, their presence alone does not predict adverse reproductive outcomes with high specificity. Consequently, their contribution to RPL is likely multifactorial and may depend on additional genetic or environmental modifiers. Because their clinical relevance remains controversial, these variants were interpreted cautiously and were not considered definitive causal findings when defining diagnostic yield. Consistent with current ESHRE guidelines and systematic reviews, our findings support the position that hereditary thrombophilia testing has limited clinical utility in RPL in the absence of additional thromboembolic risk factors. Therefore, non-selective hereditary thrombophilia testing in women with recurrent pregnancy loss is not recommended, except in cases where additional clinical risk factors are present, such as a personal or family history of venous thromboembolism [21].

Meanwhile, lower diagnostic yields in groups such as unexplained infertility (3.1%) or oligospermia (6.6%) emphasize that many infertility cases remain genetically unexplained after this limited testing approach.

NGS is increasingly used to improve diagnostic yield. Whole-exome sequencing (WES) studies in well-phenotyped cohorts of men with primary spermatogenic failure have reported diagnostic yields in the single- to low-double-digit range, depending on inclusion criteria and variant curation pipelines—for example, a cohort study of 167 men with primary infertility reported pathogenic/likely pathogenic variants in ~14% of cases [16]. Greater clinical-exome efforts likewise demonstrate ~12% molecular diagnosis in idiopathic spermatogenic failure when curated gene sets and stringent pipelines are applied [17]. Despite this progress, variants of uncertain significance remain common, underscoring the need for functional validation and international data sharing.

In female infertility, monogenic etiologies are less completely defined but are gaining more clarity. Primary ovarian insufficiency (POI), affecting ~1–2% of women under 40, has been linked to *FMR1* premutations and variants in genes such as *FOXL2*, *BMP15*, and *NOBOX*, although most cases remain idiopathic. Evidence-based curation of gene—disease associations highlights pronounced heterogeneity across pathways of folliculogenesis, hormonal regulation, mitochondrial function, and meiosis [22]. Compared with many forms of male infertility—where discrete chromosomal or Y-linked defects are often found—female infertility frequently exhibits a more polygenic and complex architecture, limiting current diagnostic yields. However, applying NGS panel testing or exome sequencing could solve a few monogenic disease cases.

Our study demonstrated that diagnostic yield varies by clinical subgroups. Men with non-obstructive azoospermia or severe oligozoospermia have the highest likelihood of identifiable genetic defects, whereas yields are lower in milder forms. In couples with recurrent pregnancy loss, parental karyotyping detects balanced chromosomal rearrangements in a small but clinically meaningful fraction, while the causal role of other variants (e.g., thrombophilia polymorphisms) remains debated [3]. From a laboratory diagnostics standpoint, these findings highlight the importance of phenotype-based test selection to maximize diagnostic yield while avoiding low-value testing in selected referral populations.

An additional limitation relates to the heterogeneity of referral indications in this tertiary-care cohort. While the overall study population included 900 patients referred for various reproductive disorders, diagnostic yield calculations were restricted to phenotypes with established indications for the evaluated tests. Patients with secondary infertility, hypogonadotropic hypogonadism, primary or secondary amenorrhea, ovarian insufficiency, and other heterogeneous reproductive conditions were therefore excluded from subgroup diagnostic yield analyses. Although broader genomic testing could potentially identify additional genetic findings in some of these patients, the aim of the present study was to evaluate the diagnostic yield of specific cytogenetic and targeted molecular tests implemented in routine clinical practice. Consequently, these findings should be interpreted within the context of the defined infertility phenotypes included in the diagnostic yield analysis.

Taken together, our findings demonstrate that diagnostic yield varies markedly by clinical subgroup within a tertiary referral setting. Genetic testing provides the greatest value in clearly defined phenotypes such as azoospermia, while yields are substantially lower in broader or less specific referral categories. These results reflect real-world referral practice and support a targeted, phenotype-guided approach to genetic evaluation in infertility. Future integration of broader genomic methods should be pursued within structured clinical and research frameworks to address unresolved cases, rather than as an indiscriminate extension of current testing strategies.

## 5. Conclusions

This five-year retrospective analysis demonstrates that, within a tertiary referral setting, phenotype-guided genetic testing—including karyotyping, Y chromosome microdeletion analysis, *CFTR* sequencing, and targeted *F2*/*F5* genotyping—yields clinically meaningful findings in a limited subset of patients evaluated for infertility and reproductive disorders. Diagnostic yield was highest among males with azoospermia, supporting the value of combined cytogenetic and targeted molecular testing in clearly defined infertility phenotypes. In contrast, substantially lower yields were observed in other referral groups, underscoring the limited utility of indiscriminate genetic testing within selected populations. Overall, these findings reflect real-world referral practice and support a targeted, phenotype-driven diagnostic strategy in infertility care, while highlighting the need for broader genomic strategies to address persistently unexplained cases in specialized care settings.

## Figures and Tables

**Table 1:** Distribution of patients’ admission reasons and their frequencies by gender.

Reproductive Disorders	Frequency in Male *n* (%)	Frequency in Female *n* (%)	Frequency in General Group *n* (%)
	*N* = 473	*N* = 427	*N* = 900
Recurrent miscarriages	107 (22.6) *	121 (28.3)	228 (25.3)
Unexplained Infertility	75 (15.9)	85 (19.9)	160 (17.8)
Azoospermia	54 (11.4)	2 (0.5) **	56 (6.2)
Oligospermia	106 (22.4)	16 (3.7) **	122 (13.6)
Hypogonadotropic hypogonadism	8 (1.7)	0 (0)	8 (0.9)
Primary/secondary Amenorrhea	0 (0)	32 (7.5)	32 (3.6)
Ovarian Insufficiency	0 (0)	7 (1.6)	7 (0.8)
Secondary infertility	100 (21.1)	137 (32.1)	237 (26.3)
Other Reproductive Disorders	23 (4.9)	27 (6.3)	50 (5.6)

* Male patients in the recurrent miscarriage group represent partners of affected women referred for couple-based karyotype evaluation. ** Female patients were not genetically tested but received genetic counseling together with their spouses, diagnosed with azoospermia and oligospermia.

**Table 2:** Distribution of genetic test findings according to reason for patient admission. * Includes results of karyotyping, Y chromosome microdeletion analysis, *CFTR* gene sequencing, and *F2*/*F5* variants testing by diagnostic subgroup.

Reproductive Disorders	Total	Prevalence *	Karyotyping (*n* = 29)	Y Chromosome Microdeletions (*n* = 6)	*CFTR* (*n* = 3)	*F2*/*F5* (*n* = 4)
Recurrent miscarriages	12	5.3%	8	-	-	4
Unexplained Infertility	5	3.1%	5	-	-	-
Azoospermia	18	33.3%	12	3	3	-
Oligospermia	7	6.6%	4	3	-	-

**Table 3:** Summary of detected genetic abnormalities (Numerical and structural chromosomal variants are written according to ISCN and grouped by type. “mos” indicates mosaicism. All sequence variants are described using HGVS nomenclature.

Category	Karyotype/Variant	Patient Count
Klinefelter Syndrome	47,XXY	9
	mos 47,XXY [28]/46,XY [12]	1
Turner Syndrome Variants	45,X	1
	mos 45,X [4]/46,XX [46]	1
	46,X,i(X)(q10)	1
	46,X,del(X)(q13.2),14pss	1
	46,X,del(X)(p11.2)	1
Sex Reversal (46,XX Male)	46,XX,inv(9)(p11q13)	1
Robertsonian Translocation	45,XY,der(13;14)(q10;q10)	3
Reciprocal Translocations	46,XY,t(2;14)(p16;q22)	1
	46,XX,t(9;12)(p23;q22)	1
	46,XX,t(8;22)(q24.1;q11.2)	1
	46,XX,t(9;15)(q12;p11.2)	1
	46,XX,t(9;12)(p24;q22)	1
Other Chromosomal Findings	mos 46,X,idic(Y)(q11.2?2) [20]/45,X [3]	1
	46,Y,add(X)(p?22)	1
	47,XY,+mar	1
	46,XY,der(15)?t(Y;15)(q12;p11.2)	1
	mos 47,XY,+mar [19]/46,XY [23]	1
*CFTR* Variants	NM_000492.4:c.[1210-33_1210-6GT [12]T [5]];[1521_1523del]	2
	NM_000492.4:c.[1210-33_1210-6GT [12]T [5]];[2052dupA]	1
*F2* Variant	NM_000506.5:c.*97G>A	3
*F5* Variant	NM_000130.4:c.1601G>A	1
Y Microdeletions	AZFc b2/b4-mediated deletion	6

## Data Availability

The data presented in this study are available on request from the corresponding author due to ethical and privacy restrictions related to the use of clinical and genetic data.

## Abbreviations

The following abbreviations are used in this manuscript:

AZF	Azoospermia factor
CBAVD	Congenital bilateral absence of the vas deferens
CFTR	Cystic fibrosis transmembrane conductance regulator
F2	Coagulation Factor II
F5	Coagulation Factor V
HGVS	Human Genome Variation Society
ISCN	International System for Human Cytogenomic Nomenclature
NGS	Next-generation sequencing
PCR	Polymerase chain reaction
RPL	Recurrent pregnancy loss
WES	Whole-exome sequencing
